# Deep Neck Infection: A Review of 130 Cases in Southern China

**DOI:** 10.1097/MD.0000000000000994

**Published:** 2015-07-13

**Authors:** Weiqiang Yang, Lijing Hu, Zhangfeng Wang, Guohui Nie, Xiaoling Li, Dongfang Lin, Jie Luo, Hao Qin, Jianhui Wu, Weiping Wen, Wenbin Lei

**Affiliations:** From the Otorhinolaryngology Hospital, Otorhinolaryngology Institute, The First Affiliated Hospital, Sun Yat–sen University (WY, LH, ZW, JL, JW, WW, WL); the Otolaryngological Department, Peking University Shenzhen Hospital, Shenzhen, Guangdong (WY, GN); Master Candidate in Department of Medical Statistics and Epidemiology, School of Public Health, Sun Yat-sen University, Guangzhou (XL); Division of Otorhinolaryngology, First People's Hospital of Foshan, Foshan (HQ); and Division of Rheumatology, The Third Affiliated Hospital of Sun Yat–sen University, Guangzhou, China (DL).

## Abstract

The study aims to present our experience of the clinical course and management of deep neck infection and try to determine if the characteristics of this kind of infection were similar between the children and adults in southern China.

Patients diagnosed with deep neck infection in the Division of Otolaryngology in the First Affiliated Hospital of Sun Yat–sen University between January 2002 and December 2011 were screened retrospectively for demographic characteristics, presenting symptoms, antibiotic therapy before admission, the history of antibiotics abuse, leucocyte count, etiology, bacteriology, disease comorbidity, imaging, treatment, complications, and outcomes.

One hundred thirty patients were included and 44 (33.8%) were younger than 18 years old (the children group), 86 patients (66.2%) were older than 18 years old (the adults group). Fever, trismus, neck pain, and odynophagia were the most common symptoms in both groups. Forty children (90.9%) and 49 adults (57.0%) had been treated with broad-spectrum antibiotic therapy before admission. Thirty one children (70.5%) and 24 adults (27.9%) had a history of antibiotics abuse. In children group, the site most commonly involved was the parapharyngeal space (18 patients, 40.9%). In adults group, the site most commonly involved was multispace (30 patients, 34.9%). In children group, the most common cause was branchial cleft cyst (5 patients, 11.4%) and the cause remained unknown in 31 patients (70.5%). In adults group, the most common cause was pharyngeal infection (19 patients, 22.2%). All of the 27 patients with associated disease comorbidity were adults and 17 were diabetes mellitus (DM). *Streptococcus viridans* was the most common pathogen in both children and adults groups. Eighty six (66.2%) underwent surgical drainage and complications were found in 31 patients (4 children, 27 adults).

Deep neck infection in adults is easier to have multispace involvement and lead to complications and appears to be more serious than that in children. Understanding the different characteristics between the children and adults with deep neck infection may be helpful in accurate evaluation and proper management.

## INTRODUCTION

Deep neck infection is defined as infection in the potential spaces and fascial planes of the neck. Although the incidence of deep neck infection has decreased significantly with the wide use of antibiotics, this condition is still common and presents a constant challenge as it may lead to lethal complications such as mediastinitis, airway obstruction, jugular vein thrombosis, pericarditis, pleural empyema, and arterial erosion.^1–3^ Moreover, an inappropriate use of antibiotics may change the clinical presentation of infections of this kind, making them elusive.^2^ Because of an especially high level of antibiotic resistance in China,^4,5^ which is attributable to the lack of strict management standards, huge profits for overprescribing, and over-the-counter availability,^6,7^ research into the clinical characteristics of deep neck infection in a Chinese population should be of epidemiological interest globally.

It is a common view that old age and diabetes mellitus (DM) are 2 important factors of deep neck infection.^8,9^ DM results in a defect in the host's immune function such as polymorphonuclear neutrophil function and cellular immunity complement activation^10^ and that increases the risk of vascular complications and the episodes of infection.^11^ The prevalence of DM among adults was about 100 times than that among children in China (9.7% and 0.1%, respectively).^12,13^ That may result in many differences in the characteristics of deep neck infection between the children and adults.

In this study, we presented our experience of the clinical course and management of deep neck infection and try to determine if the characteristics of this kind of infection were similar between the children and adults in southern China.

## METHODS

This study examined the medical records of all patients in the Division of Otolaryngology in the First Affiliated Hospital of Sun Yat–sen University who were diagnosed with deep neck infection between January 2002 and December 2011. Patients were excluded if informed consent was not given and if they exhibited superficial infections, limited intraoral abscesses, peritonsillar abscesses, cervical necrotizing fasciitis, or infections secondary to surgical neck trauma. We also excluded the patients who did not complete the treatment. Ultimately, 130 patients were included and 44 (33.8%) were younger than 18 years old (the children group). The remaining 86 patients (66.2%) were older than 18 years old (the adults group). The demographic characteristics, presenting symptoms, antibiotic therapy before admission, leucocyte count, etiology, bacteriology, disease comorbidity, imaging, treatment, complications, and outcomes of each case were reviewed. The history of antibiotics abuse of the patients was also evaluated by telephone follow-up and letter follow-up. Patients who were given antibiotics by physicians to cure a common cold more than 3 times were determined to have a history of antibiotics abuse. In accord with the previously published literature,^8,14^ the site of infection was categorized in the following spaces: submandibular, parapharyngeal, parotid, masticatory, carotid, retropharyngeal, prevertebral, anterior cervical, posterior cervical, and pretracheal space. Patients who had infection in 2 or more spaces were classified as having multispace involvement. Information that characterized a patient's infection was identified on the basis of clinical symptoms, imaging studies, needle aspiration, and surgery.

This study has been approved by the medical ethics committee of the First Affiliated Hospital of Sun Yat–sen University and performed in accordance with the ethical standards laid down in the 1964 Declaration of Helsinki.

All descriptive data were reported in percentages. Statistical evaluations were performed with a 2-sided *t*-test corrected for inequality of variances and degrees of freedom. Fisher exact test and *χ*^2^ test were used to compare the categorical variable. SPSS (13.0, SPSS Inc, Chicago, IL) were used to analyze the data and a *P*-value of <0.05 was considered statistically significant.

## RESULTS

### Patient Characteristics

**FIGURE 1:**
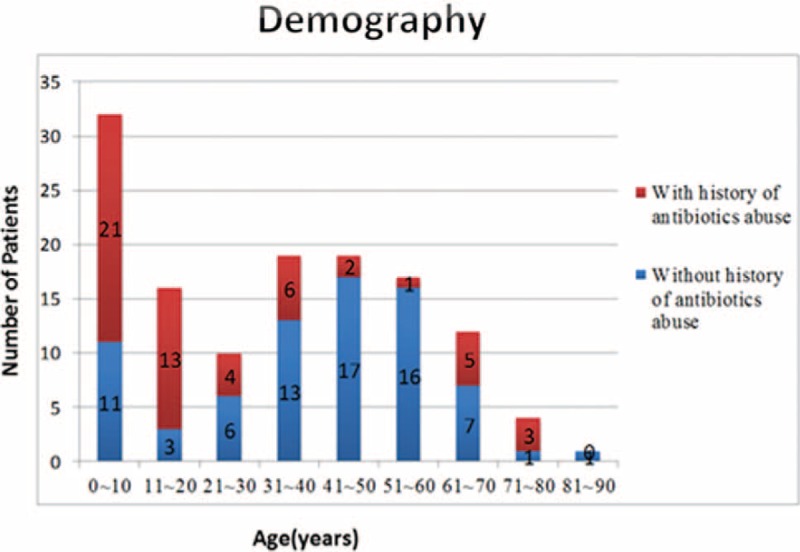
Age distribution of patients and history of antibiotics abuse.

Of the 130 patients, 79 (60.8%) were male and 51 (39.2%) were female, ranging in age from 15 day to 82 years with a mean age (± SD) of 32.9 (± 22.8) years. The single largest age category was from birth to 10 years (32 cases, 24.6%) as shown in Figure 1. In children group, 21 (47.7%) were male and 23 were female. In adults group, 58 (67.4%) were male and 28 were female.

Fever, trismus, neck pain, and odynophagia were the most common symptoms in both children and adults groups (Table 1). Fever was more frequent in the children than in adults (93.2% vs. 45.3%, *P* < 0.001). Neck pain was found in 54.5% children and 74.4% adults (*P* = 0.02) while odynophagia was found in 47.7% children and 66.3% adults (*P* = 0.04). Forty children (90.9%) and 49 adults (57.0%) had been treated with broad-spectrum antibiotic therapy before admission (*P* < 0.001). Thirty one children (70.5%) and 24 adults (27.9%) had a history of antibiotics abuse (*P* < 0.001) (Table 2). The data of leucocyte count were available in 123 patients (41 children and 82 adults). The reference range of leucocyte count was 4.0–10.0 × 10^9^ cells /L. The leucocyte count was abnormal (leucocyte count <4.0 × 10^9^ cells /L or >10.0 × 10^9^ cells /L) in 36 (87.8%) children and 55(67.1%) adults (*P* = 0.013). Statistical analysis showed that mean leucocyte and lymphocyte counts in children group were both significant higher than in adults group (*P* = 0.012 and *P* = 0.001, respectively) (Table 3).

**TABLE 1:**
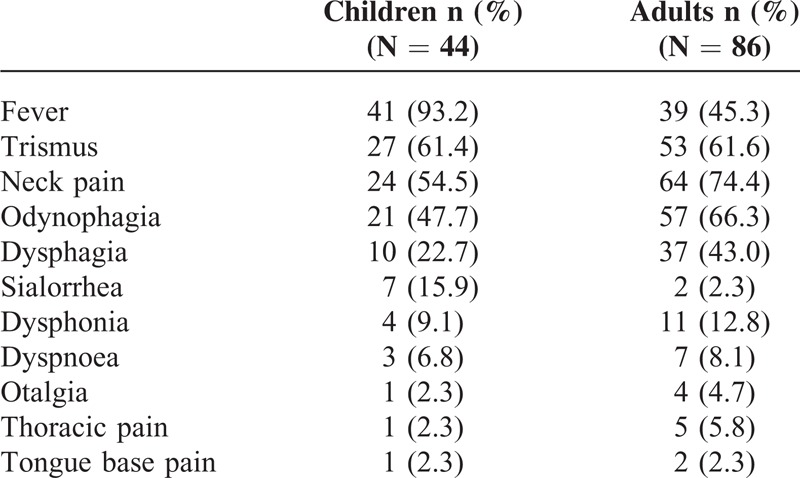
Presenting Symptoms of Deep Neck Infection

**TABLE 2:**
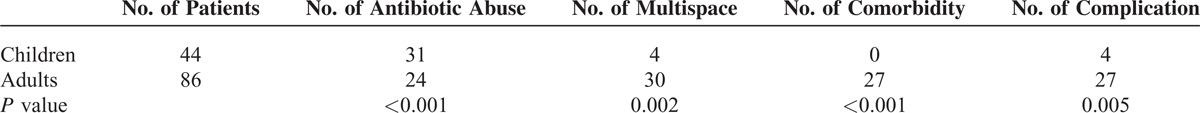
Comparison of Children and Adults

**TABLE 3:**
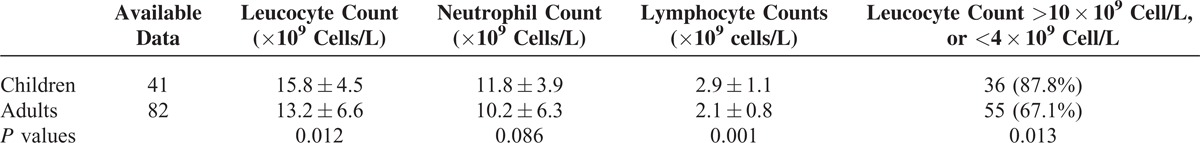
The Laboratory Data of Deep Neck Infection

### Site of Infection

Computed tomography scans (CT scans) with contrast enhancement were included in routine investigation. MRI scans with contrast enhancement were performed in 11 (8.5%) patients. Orthopantograms (OPG) of the mandible and sonography of the neck were performed in 13 and 15 patients, respectively. As Table 4 shows, the site most commonly involved in children group was the parapharyngeal space (18 patients, 40.9%), followed by submandibular space in 8 patients (18.2%), the retropharyngeal space in 5 patients (11.4%). In adults group, the site most commonly involved was multispace (30 patients, 34.9%), followed by parpharyngeal space in 17 patients (19.8%), the submandibular space in 12 patients (14.0%). The adult patients developed multispace infection more often than the children (*P* = 0.002) (Table 2).

**TABLE 4:**
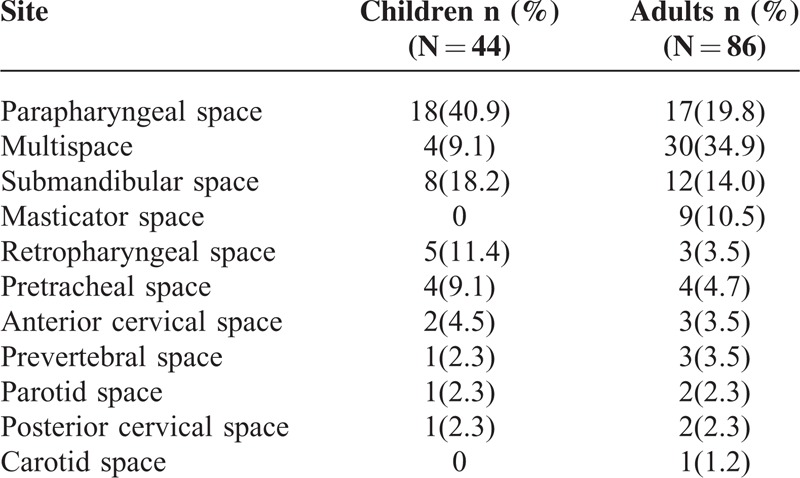
Distribution of the Sites of Deep Neck Infection

### Etiology

The causes of deep neck infection were identified in 58 cases (44.6%). The cause remained unknown in 72 patients (55.4%). In children group, the most common cause was branchial cleft cyst (5 patients, 11.4%) and the cause remained unknown in 31 patients (70.5%). The data in the adults were pharyngeal infection (19 patients, 22.2%) and 41 patients (47.7%), respectively, as recorded in Table 5. An esophageal foreign body was observed in 9 cases, accounting for 4 of the 8 cases of retropharyngeal space infections and 4 of the 12 cases of descending mediastinitis.

**TABLE 5:**
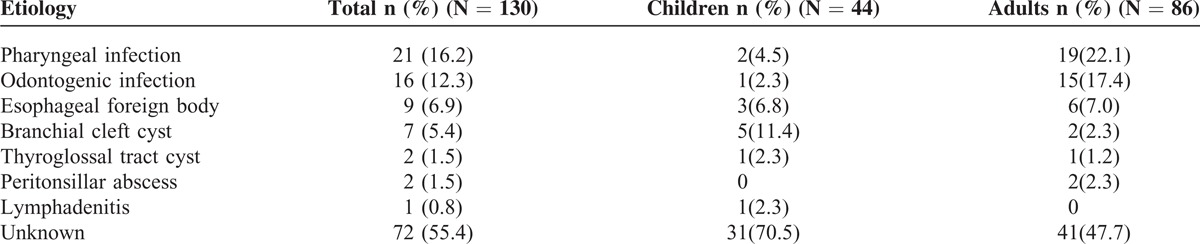
Etiology of Deep Neck Infection

### Disease Comorbidity

All of the 27 patients (20.8%) with associated disease comorbidity were adults (*P* < 0.001) (Table 2). Among them, 17 had diabetes mellitus (DM). Nasopharyngeal carcinoma after radiotherapy was found in 5 patients, of which 3 had masticatory space infection. There were 4 patients with uremia or chronic renal insufficiency, 2 patients with systemic lupus erythematosus under steroid therapy, 1 patient with acute myeloid leukemia, and 1 patient with cirrhosis. Three patients had 2 associated diseases. Patients with associated disease were older (49.4 ± 11.9 years old vs. 28.6 ± 23.0, *P* < 0.001) and apparently were at an elevated risk of multispace infections (10/27 vs. 24/103) and have a longer hospital stay (19.4 days vs. 13.8 days).

### Bacteriology

Samples for bacterial culture were collected from 70 patients (53.8%), and bacteria were found in 49 samples. *Streptococcus viridans* was the most common pathogen in both children and adults groups, found in 10 patients (10 of 20 positive samples, 50%) and 14 patients (14 of 29 positive samples, 48.3%), respectively. Among these 49 positive samples, polymicrobial infections were found in 6 (12.2%), 2 of them were children and the other 4 were adults. Of the 6 patients with *K. pneumoniae*, 5 were adults and 3 had DM. The culture results are shown in Table 6.

**TABLE 6:**
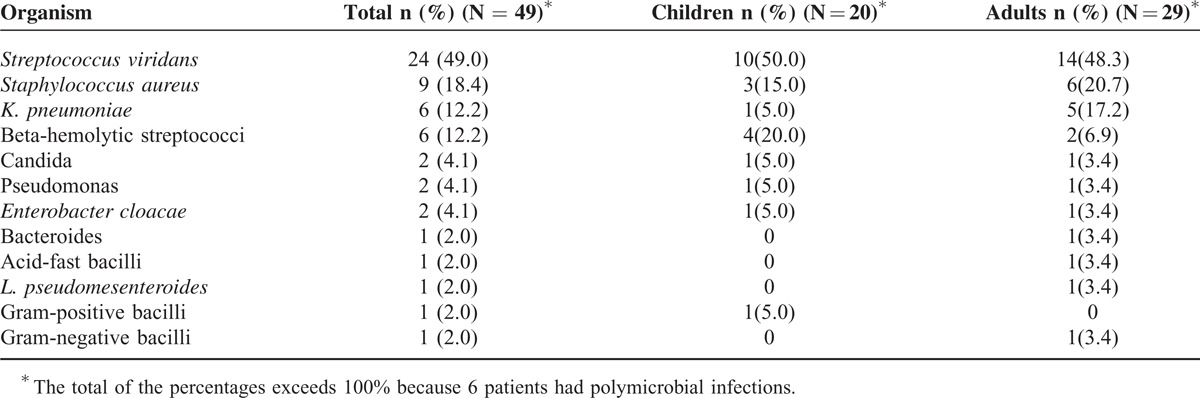
Organisms Detected in Pus Culture of Patients With Positive Cultures

### Treatment and Outcomes

All 130 patients were treated with intravenous antibiotics on admission, and of these, 86 (66.2%) underwent surgical drainage. It was more often to undergo surgical drainage in children group than in adults group (84.7% vs. 57.0%, *P* = 0.034). Repeated needle aspiration was undergone in 1 child and 8 adults, respectively. The other 6 children and 29 adults received only conservative management in addition. The duration of hospital stay ranged from 1 to 62 days, with a mean of 11.9 ± 9.8 days in children group and 16.5 ± 13.4 days in adults group (*P* = 0.026). One patient, a 55-year-old man without any associative diseases died of septic shock and multiple organ dysfunction syndromes after an upper gastrointestinal perforation and acute diffuse peritonitis.

### Complications

The complications of deep neck infection are shown in Table 7. Complications were found in 31 patients (4 children, 27 adults), with a mean age of 41.3 ± 18.5 years. Of the 4 patients of children with complications, 3 had airway obstruction and 1 had skin defeat. Of the 27 adults with complications, 12 had multiple complications and 11 were multispace involved. Airway obstruction was experienced by 17 patients, of which 9 underwent either tracheotomy or endotracheal intubation. Of the 12 cases of mediastinitis, 4 were caused by esophageal foreign body, 6 were multispace infections, and only 1 involved associated disease.

**TABLE 7:**
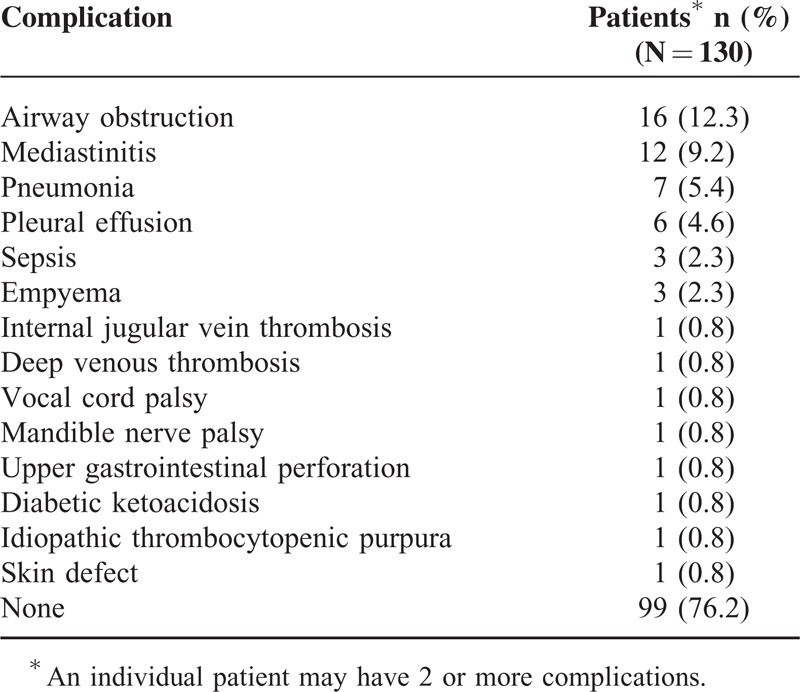
Complications of Deep Neck Infection

## DISCUSSION

Huang et al^8,15^ and Lee et al^3^ indicated that old patients with DM were susceptible to deep neck infection. An age-related decline in neutrophil function, including impairment of neutrophil phagocytosis and bactericidal ability in healthy individuals, may contribute to increased susceptibility to bacterial infections in the elderly population.^16,17^ In DM patients, hyperglycemia may impair several mechanisms of humoral host defense, such as varied neutrophil functions: adhesion, chemotaxis and phagocytosis and result in predisposition to infection.^10^ Most previous studies^1,3,8,18,19^ reported that children are a relatively low proportion of their patients with deep neck infection. We found, however, that the proportion of patients who were under 18 years of age were high (44 cases, 33.8%), and none of them had DM or other associated diseases. We analyzed that 1 of the factors for our higher proportion of children may be related to a history of antibiotics abuse and the antibiotic resistance. As previous reports revealed, the frequency of common colds in children per year was about 2–3 times than that in adults (6–8 vs. 2–4 times on average).^20–22^ According to a survey in Beijing Children's Hospital, more than 98% of children patients in the Outpatient Department who were diagnosed with common cold were given antibiotics by physicians.^23^ Another survey to about 1500 students’ parents showed that the rate of parental self-medication for the children was 59.4%.^24^ These situations may together contribute to a more severe antibiotics abuse in Chinese children. In our data, the rate of a history of antibiotics abuse in children was 70.5% which was greatly higher than 27.9% in adults (*P* < 0.001) and the rate of previous broad-spectrum antibiotic therapy in children is 90.9% which was higher than 57% in adults (*P* < 0.001). Previous antibiotic use is correlated with higher recovery of resistant organisms^25,26^ and increased incidence of β-lactamase-producing bacteria.^27^ Ultimately, the resistance had an effect on the incidence of deep neck infection.^28^

Male patients (67.4%) obviously predominated in adults, as also found by Marioni et al^29^ and Eftekharian et al,^9^ while it showed an equal distribution in the ratio between the sexes in children group. Fever, trismus, neck pain, and odynophagia were the most common symptoms in both children and adults groups as Table 1 shows. Neck pain and odynophagia were found less frequent in children. The reason may be because the symptoms were not easy to be evaluated in children and that resulted in a more subtle presentation.^30^ Our data showed the count of the leucocyte in children group is high (15.8 ± 4.5 × 10^9^ cells/L) and in adults group is low (13.2 ± 6.6 × 10^9^ cells/L). The available data of leucocyte count in children group included 9 infants (age < 1 year) and 32 older children (1 year <age<18 years). The mean leucocyte count of the 9 infants and the 32 older children was 19.2 × 10^9^ and 14.9 × 10^9^ cells/L, respectively. It has been recognized that adults have lower mean leucocyte count than children and infants. We supposed the advantage of the leucocyte in children group to adults group was, in fact, the advantage of the normal range.

The value of imaging study in deep neck infection is delineating the exact anatomical extent and detecting complications so that correct localization of the involved space for timely incision and drainage can be made.^31^ CT scans with contrast enhancement can detect cellulitis and abscesses and is also quite helpful for marking abscesses by the rim enhancement, estimating the space involvement, and indicating complications.^32–34^ Therefore, we consider CT scans with contrast enhancement as routine investigation of deep neck infection.

Most studies^35,36^ reported the retropharyngeal space as the most common involved space in children, but we found the parapharyngeal space the most common involved in children group (18 of 44 patients, 40.9%). Infections in the peritonsillar, submandibular, masticatory, and parotid space can usually spread to the parapharyngeal space.^8,14^ Multispace infection was found in 30 patients (34.9%) in adults and in 4 patients (9.1%) in children (*P* = 0.002). Adults were easier to get multispace infection than children; this might relate to a higher incidence of disease comorbidity in adults. Patients with disease comorbidity tend to have poorer defense against infections and thus result in higher rates of more severe infection in the form of multispace infections.^3^

A large number of cases with unknown etiology (72 patients, 55.4%) were noted in our study; this may be attributed to the 70% of broad-spectrum antibiotic therapy before admission. A higher rate of unknown etiology in children (31 of 44 patients, 70.5%) may also relate to the fact that the children have a more subtle presentation in that they are seldom able to describe their symptoms or cooperate with the physical examination, and their oropharynx is frequently difficult to examine because of its small size.^28^ Some other studies implicate odontogenic infections as the leading cause of deep neck infection.^3,8,37,38^ In our series, the most common cause of deep neck infection was pharyngeal infection in adults, as also reported by Boscolo-Rizzo et al. Five patients of branchial cleft cyst were noted as the most common etiology in children; this can be easily explained by the high rate of unknown etiology. An esophageal foreign body was observed in 9 cases, accounting for 4 of the 8 cases of retropharyngeal space infection and 4 of the 12 cases of mediastinitis. The reason is that the retropharyngeal space is posterior to the pharynx and esophagus and extends from the base of the skull down into the mediastinum,^33^ so a foreign body in the esophagus can easily fall into the retropharyngeal space and lead to mediastinitis.

No bacteria growth was noted in 30% of available samples and that was likely due to of high rate of broad-spectrum antibiotic therapy before admission and the use of intravenous antibiotics before surgical drainage. As other studies report,^8,37,38^ the organism most commonly isolated was *Streptococcus viridans* (24 cases, 49% of positive samples). It is widely believed that *Streptococcus viridans* is always bound up with odontogenic infections,^8,37^ and the fact that only 2 out of 24 patients with *Streptococcus viridans* positive were related to odontogenic infections in our study, whereas half were idiopathic, may suggest that a large proportion of cases with no known primary source may, in fact, have been odontogenic in origin.

Associated disease was found in 27 cases (20.8%) and all of them were adults. DM (17 cases, 63.0%) was the most common. Because *K. pneumoniae* was the top pathogen in patients with DM (3 cases, 17.6%), antibiotic coverage against this organism should be emphasized in such patients.^3^ *K. pneumoniae* infection should always be considered in DM patients, regardless of whether they are adults or children.^39^ As expected, patients with associated disease were older (*P* < 0.001) and apparently were at an elevated risk of multispace infections (10/27 vs. 24/103) and have a longer hospital stay (19.4 vs. 13.8 days). Therefore, more attention should be paid to the patients with associated diseases.

All 130 patients were treated with intravenous antibiotics on admission. Empirical parenteral antibiotics were started before the culture results become available and then tailored to the culture results when available. The current literature^40–42^ suggests deep neck infection in children can often be successfully managed with medical therapy alone but life-threatening complications may occur.^41^ However, we believe incision and drainage as the gold standard for the majority of pediatric deep neck abscesses. Our data showed a high rate surgical drainage in children group (37 patients, 84.2%) and a low incidence of complications (4 patients, 9.1%). The duration of hospital stay in children was shorter than that in adults (*P* < 0.05). When dealing with deep neck infection in adults, we should pay more attention to the comorbidity, particularly to DM patients’ blood glucose level.^3^ We believe the primary treatment on deep neck infection in adults is surgical drainage, but needle aspiration and high-dose intravenous antibiotics might be sufficient in selected cases with a good response to antibiotics, a small abscess, and no lethal complications. We successfully treated 29 (33.7%) cases only by intravenous antibiotics and 8 cases (9.3%) by repeated needle aspirations.

Despite the widespread use of antibiotics, deep neck infection can still lead to life-threatening complications such as descending mediastinitis, airway obstruction, jugular vein thrombosis, pericarditis, pleural empyema, and arterial erosion. In our study, complications were noted in more adults patients than in children patients (27/86 vs. 4/44, *P* < 0.001) which was shown in Table 2. The reason may be partly related to the fact that the adults group had more patients with multispace infection and all the 27 patients with comorbidity were adults. Of the 3 patients with sepsis, 1 (mortality rate, 0.77%) died of septic shock and multiple organ dysfunction syndrome after upper gastrointestinal perforation and acute diffuse peritonitis.

## CONCLUSIONS

Deep neck infection in adults is easier to have multispace involvement and lead to complications and appears to be more serious than that in children. Understanding the different characteristics between the children and adults with deep neck infection may be helpful in accurate evaluation and proper management.
